# Translation, cross-cultural adaptation, and psychometric evaluation of the Swedish Work-Life Balance Scale

**DOI:** 10.1186/s40359-026-05752-3

**Published:** 2026-10-09

**Authors:** Frida Degerstedt, Susanna Bellander, Joakim Ingrell, Peter G Tedeholm, Therese Nordin, Elin Granholm Valmari

**Affiliations:** 1 Department of Community Medicine and Rehabilitation, Umeå University, Umeå, Sweden; 2 School of Police Sciences, Södertörn University, Södertörn, Sweden; 3 Department of Police Work, Malmö University, Malmö, Sweden; 4 Department of Physiology and Pharmacology, Karolinska Institutet, Solna, Sweden

**Keywords:** Life balance, Occupational balance, Reliability, Validity, Workers’ health, Work-life

## Abstract

**Background:**

Work-life balance concerns individuals’ subjective experience of how well work and non-work activities fit together. Work-life balance has been associated with, for instance, psychological health and other occupational outcomes. The four-item Work-Life Balance Scale provides a brief measure of this construct, but no culturally adapted and psychometrically evaluated Swedish version has previously been available. This study aimed to translate and cross-culturally validate the scale for use in Sweden and to conduct a psychometric evaluation.

**Methods:**

The scale was translated and cross-culturally adapted in accordance with standard guidelines. The process included preparation, two independent forward translations, reconciliation, two independent back translations, equivalence review, cognitive debriefing with eight Swedish-speaking participants, proofreading, and finalization. Psychometric evaluation was conducted using cross-sectional survey data from 215 students across both the police and the prison and probation service education. Internal consistency was examined using Cronbach’s alpha, McDonald’s omega, and corrected item-rest correlations. The proposed one-factor structure was evaluated using confirmatory factor analysis. Convergent validity was assessed in relation to occupational balance. Moreover, construct validity through hypothesized associations was examined with depressive and anxiety symptoms.

**Results:**

Complete Work-Life Balance Scale data were available for 215 participants. The mean scale score was 3.85 (SD = 0.85) out of 5. Internal consistency was found to be good according to both Cronbach’s and McDonald’s ω. However, the reverse-worded item showed a lower corrected item-rest correlation than the other three. The one-factor model demonstrated mixed but generally acceptable fit, as confirmed by the confirmatory factor analysis. Again, the reverse-worded item showed the lowest loading. Work-life balance correlated positively with occupational balance, indicating convergent validity, while being negatively associated with depressive symptoms and anxiety symptoms, as hypothesized.

**Conclusions:**

The Swedish Work-Life Balance Scale demonstrated promising initial psychometric properties, including good internal consistency and convergent validity. Additional studies should evaluate test-retest reliability, measurement invariance, responsiveness, and predictive validity in broader Swedish working populations.

## Introduction

Work may cause illness [1], and paradoxically also reduction in work ability, i.e., the physical and cognitive ability to perform at work [2, 3]. However, workers’ health may also be affected by the interaction between work and private life [4, 5], in which conditions at the intersection of these domains may create or intensify conflict between occupational and personal demands [5, 6]. Experiencing work-life balance also influences other occupational outcomes, such as organizational commitment [7], job satisfaction [8] or retention [9]. Consequently, the relationship between work and non-work life has received considerable attention in organizational psychology, often focusing on e.g., gender differences, flexible work arrangements, policies and practices [10, 11].

Many different theoretical perspectives on work-life balance have been developed to explain the interaction between work and private life [12]. One of these is the work-family conflict theory that describes how incompatible demands arising from work and family roles may negatively affect occupational functioning, family life, and health [13]. Long working hours have, for example, been associated with greater conflict between work and family life [14]. In contrast, work-family enrichment theory emphasizes that resources acquired through participation in one role may enhance experiences and functioning in another role [15, 16]. The spillover-crossover model provides another perspective on how experiences originating in one domain may affect other aspects of life. Spillover refers to the transfer of experiences, emotions, or thoughts between work and private life domains within the same individual, whereas crossover refers to their transmission between individuals, such as from a partner or family member [17, 18]. However, the conceptual definition of work-life balance is distinct from theories of work-family conflict, spillover, and work-life enrichment. For instance, work-life conflict focuses on incompatible demands between roles, whereas work-life enrichment focuses on how resources gained in one domain can benefit another. In contrast, work-life balance refers to individuals’ overall and subjective appraisal of how well work and non-work activities fit together in relation to their current priorities and opportunities for growth [19]. Such a balance does not necessarily imply an equal allocation of time or effort across domains, as individuals may perceive balance even with unequal role investments provided that these investments align with their current priorities [12].

Perreault and Power [20], have also argued that although work-life balance models in general often address how, e.g., time and energy are distributed between work and private life as the two significant domains in an adult’s life, theories within organizational psychology have often been developed primarily from an organizational perspective, with private life treated as secondary. A more individually oriented perspective has therefore been proposed, emphasizing the dynamic interplay between work and non-work domains and individuals’ subjective evaluations of this relationship [20]. One such individually oriented concept, rooted in occupational science and occupational therapy, is occupational balance, which concerns individuals’ subjective experience of having an appropriate amount and variety of everyday activities in life, regardless of their relation to work or private life [21]. Thus, occupational balance encompasses participation across multiple activities in everyday life, whereas work-life balance focuses more specifically on the perceived relationship between work and non-work activities [22].

As there are many theories explaining work-life balance in different ways [12], there are also different ways of measuring work-life balance due to differences in the definition of the concept itself [23, 24]. Brough et al. [25], developed a concise four-item measure of work-life balance based on individuals’ global subjective appraisal of the balance between work and non-work activities. The scale, i.e., the Work-Life Balance Scale, was designed to be applicable regardless of marital or parental status. In contrast to conflict measures, which assess the extent to which demands in one domain interfere with participation or functioning in another, the scale asks respondents to evaluate the perceived balance itself. In the original validation study, its one-factor structure and internal consistency were supported across four heterogeneous samples of workers in Australia and New Zealand. Work-life balance was also associated in the expected directions with work demands, job and family satisfaction, turnover intentions, and psychological strain, with several associations replicated longitudinally [25].

The relationship between work-life balance and mental health is also relevant to the evaluation of the scale’s construct validity, especially if the scale is intended for use within work-life health studies. The original validation study also found that higher work-life balance was associated with lower psychological strain, both cross-sectionally and longitudinally [25]. Depressive and anxiety symptoms represent more specific indicators of psychological distress and would therefore be expected to show negative associations with perceived work-life balance. This was shown in prior studies using other work-life balance measures [26]in older age groups [27], as well as younger, such as those born in the late 90s and early 20s [28], or college students [29].

Although the original Work-Life Balance Scale [25] demonstrated promising psychometric properties, it has not been translated and culturally adapted for Swedish-speaking populations. Furthermore, its psychometric properties have not previously been evaluated in a Swedish context. This limits the availability of a concise measure for assessing subjective work-life balance in Swedish research and practice when conducting large work-life health studies. A systematic cross-cultural adaptation and psychometric evaluation are therefore required. Accordingly, the aim of the present study was to translate and cross-culturally adapt the Work-Life Balance Scale for use in Sweden for Swedish speakers and to evaluate the psychometric properties of the Swedish version. The following research questions were addressed:

- What are the item-level characteristics of the Swedish Work-Life Balance Scale in the study sample?
- Does the Swedish Work-Life Balance Scale demonstrate adequate internal consistency?
- Does the Swedish version support the original one-factor structure of the Work-Life Balance Scale?
- Does the Swedish Work-Life Balance Scale show evidence of convergent validity through its association with occupational balance?
- Does the Swedish Work-Life Balance Scale demonstrate validity through theoretically expected associations with symptoms of depression and anxiety?

## Methods

### Study design

**Fig. 1:**
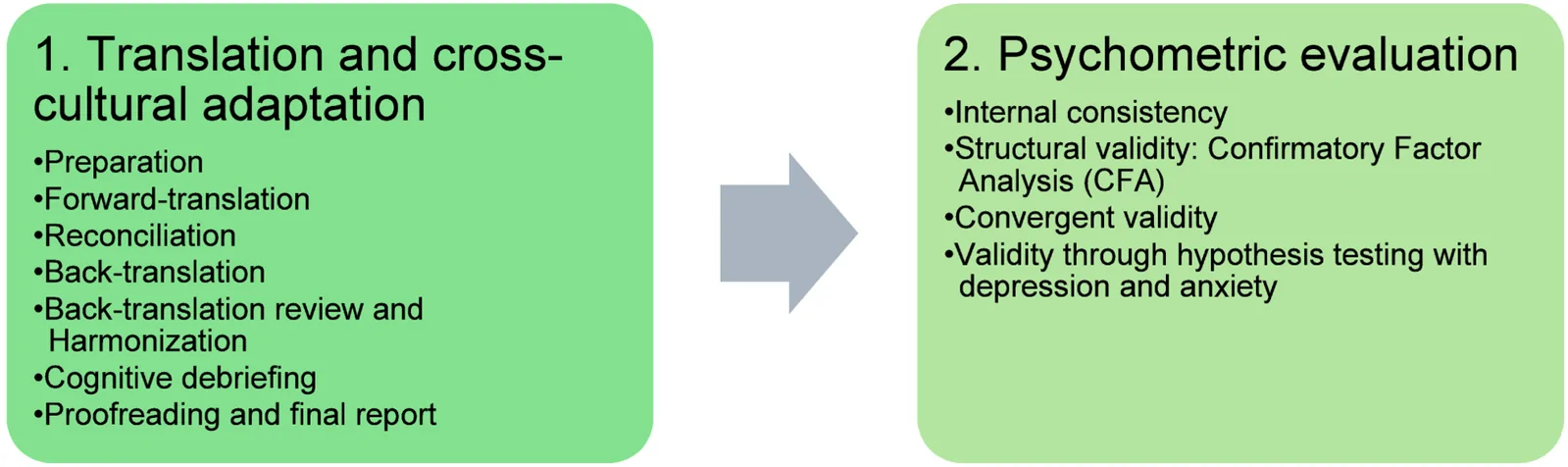
Translation, cross-cultural validation, and psychometric evaluation

This study used a methodological, cross-sectional design to translate, culturally adapt, and evaluate the psychometric properties of the Swedish version of the Work-Life Balance Scale. The study formed part of a larger research project examining health, work ability, and sustainable working life among public emergency and security personnel from professional education through the transition into working life. The study comprises two phases: (1) translation and cross-cultural adaptation of the four-item Work-Life Balance Scale, including cognitive debriefing with eight participants, and (2) psychometric evaluation of the Swedish version in a sample of students enrolled in police education, and prison and probation service education (Fig. 1). The study was approved by the Swedish Ethical Review Authority (Reg. No. 2025-00355-01) and was conducted in accordance with the ethical principles of the Declaration of Helsinki and applicable Swedish laws and regulations. For instance, all participants received written information about the study and provided informed consent prior to participation.

### Translation and cross-cultural adaptation

The authors translated the Work-Life Balance Scale from English into Swedish, and none of them is bilingual. The authors then cross-culturally adapted it in accordance with the recommended process, following ISPOR (International Society for Pharmacoeconomics and Outcomes Research) guidelines for cross-cultural validation of instruments [30]. No professional translation service was used, as all authors considered themselves sufficient in English to translate a relatively short instrument.

#### *Preparation*

As a first step, the last author contacted the instrument developer to ask for permission to translate the work-balance measure into Swedish in Sweden. The last author also coordinated the translation and cross-cultural adaptation process of the instrument. During the preparation phase, the translation procedure, responsibilities of the research team members, and intended use of the Swedish version were established.

#### *Forward translation*

Two independent forward translations from English into Swedish were produced by the third and last authors. Both forward translators were native Swedish speakers proficient in English. Before beginning the translations, the translators received information regarding the constructs assessed by the instruments, the intended target population, and the purpose of the translation. The translators were instructed to prioritize conceptual and culturally appropriate equivalence over literal, word-for-word translation.

#### *Reconciliation*

The two forward translators compared their independently produced Swedish versions item by item. The authors discussed differences in wording, meaning, terminology, and cultural appropriateness until they reached consensus, resulting in one reconciled Swedish version. When the authors could not reach consensus, the second author served as an additional reviewer and helped resolve discrepancies.

#### *Back-translation*

The reconciled Swedish version was then independently translated back into English by the fourth and fifth authors. The back-translators were not involved in the forward-translation process and were instructed to produce relatively literal translations of the Swedish wording. The purpose of the back translations was to identify discrepancies, ambiguities, or unintended changes in meaning rather than to recreate the wording of the original instruments.

#### *Back-translation review and harmonization*

The first author reviewed both back translations and compared them with the reconciled Swedish versions and the original English instrument. The first author examined differences between the back translations and the original versions to identify possible shifts in meaning or conceptual inconsistencies. Where necessary, the Swedish wording was revised to improve equivalence with the original instruments. The first author subsequently conducted a harmonization review in which the preliminary Swedish versions, the original English versions, and the two back translations were compared across all items, instructions, and response alternatives. The authors paid particular attention to conceptual equivalence, consistent terminology, comprehensibility, and the appropriateness of the wording for the Swedish context. This process produced a preliminary Swedish version for cognitive debriefing.

#### *Cognitive debriefing*

The preliminary Swedish versions were evaluated through cognitive debriefing with eight Swedish-speaking participants representing relevant occupational groups and potential users of the instrument. Of the eight participants, five were women aged 42 to 48, and three were men aged 51 to 57. Four were educated police officers, while three were civilians working within the police authority. As the Work-Life Balance Scale will be used in contexts beyond policing, one participant working as a public order officer *(‘ordningsvakt’*, i.e.,* a Swedish profession in which specific security personnel receive education from the Police Authority and work under regulated police mandate)* was also included.

The cognitive debriefing was led by the second author. The interviewer documented and summarized participants’ interpretations and suggested revisions. Participants completed the instrument and were asked about their understanding of the instructions, items, key terms, and response alternatives. They were also asked to identify wording that was unclear, ambiguous, difficult to interpret, or culturally unacceptable. Then, the first and last authors discussed the suggestions and recommended changes according to the participants’ feedback.

#### *Proofreading and final report*

Final proofreading was conducted by the second author. After this step, the first and last authors met to discuss specific wording and whether changes were needed, noting discrepancies between the original English scale and the Swedish translation, which suggested revisions to the Swedish translation. This procedure is further reported in the results section

### Psychometric evaluation

#### Participants and data collection

Participants were recruited from Swedish police education programs at three universities and from an educational program within the Swedish Prison and Probation Service. Data from police students were collected during the autumn semester of 2025 and the spring semester of 2026. Data from students in the prison and probation service were collected during spring 2026.

The questionnaire was administered electronically using Research Electronic Data Capture (REDCap) by sending the link to students via their education platform, and at one university by physically visiting classrooms and asking students to participate. Participation was voluntary, and responses were collected after participants had received study information and provided informed consent. A total of 232 participants completed the background questionnaire, but complete responses to all four items of the Work-Life Balance Scale were available only for 215 participants. These participants constituted the primary sample for the psychometric analyses.

### Measures

**The Work-Life Balance Scale** is a four-item self-report measure that assesses individuals’ subjective perceptions of the balance between work and non-work activities [25]. Because we collected data on students, we initiated the questionnaire with the following sentence: ‘If you are a student, work means your studies’. Items are rated on a five-point Likert-type response scale ranging from 1 (strongly disagree), 2 (disagree), 3 (neutral), 4 (agree), to 5 (strongly agree). One item (item 2) is negatively worded and should be reverse-coded before the scale score and psychometric statistics are calculated. A mean score was calculated across the four items, with higher scores indicating higher perceived work-life balance. A scale score was calculated only for participants who had answered all four items.

**The Occupational Balance Questionnaire (OBQ-11)** [21, 31] was used to examine convergent validity. The instrument assesses individuals’ perceived balance across everyday activities and their satisfaction with the amount and variety of those activities. The instrument consists of 11 items using a Likert scale with four options, 0 (completely disagree), 1 (tend to disagree), 2 (tend to agree), 3 (completely agree). Scores range from 0 to 33, with higher scores indicating greater occupational balance. It is a psychometrically sound instrument, initially created for the Swedish population [21, 31], but has now been translated into several other languages e.g. Turkish, Norwegian, and Persian [32–34], and has also been used in research and clinical work for a decade [21, 31]. The OBQ-11 was selected because occupational balance is conceptually related to, but more detailed than work-life balance, focusing on different aspects of activities in life, such as variety or time factors [22].

**The Patient Health Questionnaire-9 (PHQ-9)** [35] was used to assess depressive symptoms during the previous two weeks. The instrument consists of nine items rated from 0 (not at all) to 3 (nearly every day), and has been used clinically and within research in a Swedish context since 2010, showing a stable factor structure and high internal consistency [36–38]. The PHQ-9 is also suggested by the Swedish Agency for Health Technology Assessment and Assessment of Social Services [39] as a suitable measure for assessing depression. Item scores were summed to produce a total score ranging from 0 to 27, with higher scores indicating more severe depressive symptoms. A total score was calculated only when all nine items had been completed [35]. The PHQ-9 has been translated into several languages and is a well-validated, reliable measure of depression, used in both research and clinical settings [40].

**The Generalized Anxiety Disorder-7 (GAD-7)** [41] was used to assess symptoms of general anxiety disorder during the previous two weeks. It is a commonly used instrument for assessing anxiety within the Swedish context, used both clinically and within research ([38, 42, 43]. The instrument consists of seven items rated from 0 (not at all) to 3 (nearly every day). Item scores were summed up to produce a total score ranging from 0 to 21, with higher scores indicating more severe anxiety symptoms. A total score was calculated only when all seven items had been completed [41]. The GAD-7 has been translated into several languages and is a validated and reliable measure of anxiety, used in research and clinical practice [44, 45], even though there have been recent criticism on the one-factor structure within an adolescent population [46]

### Statistical analysis

Descriptive statistics were calculated for participant characteristics and the Work-Life Balance Scale items. Categorical variables were summarized using frequencies and percentages. Continuous variables were summarized using means, standard deviations, medians, and ranges, as appropriate. Item-level analyses included means, standard deviations, medians, minimum and maximum values, skewness, and kurtosis.

The negatively worded Work-Life Balance Scale item was reverse-coded before calculating the scale score, internal consistency estimates, and conducting CFA. The total scale score was expressed as the mean of the four items, with a possible range from 1 to 5. Floor and ceiling effects were considered present when more than 15% of participants obtained the lowest or highest possible score, in line with quality criteria for measurement properties [47].

Internal consistency was examined using Cronbach’s alpha [48], complemented by McDonald’s omega [49] as the sample size is rather small [50]. Corrected item-rest correlations and changes in alpha and omega if an item was deleted were also examined. Coefficients of at least 0.70 were considered indicative of acceptable internal consistency in line with Terwee et al., [47].

Structural validity was evaluated using CFA. A one-factor model corresponding to the structure proposed in the original validation study was specified. Because the items were measured using ordered response categories, the items were treated as ordinal indicators and the model was estimated using diagonally weighted least squares (DWLS) with robust standard errors and a mean-adjusted scaled test statistic. Model fit was evaluated using the chi-square test, the standardized root mean square residual (SRMR), SRMR ≤ 0.08, the root mean square error of approximation (RMSEA) with its 95% confidence interval, the comparative fit index (CFI), and the Tucker-Lewis index (TLI) with ≥ 0.95. The magnitude and statistical significance of standardized factor loadings were also examined to evaluate how strongly each item represented the latent work-life balance factor. Complete-case analysis was used for the CFA. Model fit was not based on any single cutoff value.

Convergent validity was evaluated by calculating the Pearson correlation between the Work-Life Balance Scale and the OBQ-11. Pearson correlations were used because scale scores were treated as approximately continuous, and no severe distributional problems (kurtosis, skewness, or outliers) were identified. A positive correlation was hypothesized because a higher work-life balance was expected to be associated with a higher occupational balance. Convergent validity was considered supported if the correlation with occupational balance was positive and at least moderate in magnitude.

Validity was further evaluated through hypothesis testing. Based on previous evidence in the original study linking work-life balance with psychological strain [25], it was hypothesized that higher Work-Life Balance Scale scores would be moderately and negatively associated with PHQ-9 and GAD-7 scores. The mental health hypotheses were considered supported if correlations with the PHQ-9 and GAD-7 were negative and of at least moderate magnitude. Pairwise deletion was used in the correlation analyses. Correlation coefficients were interpreted according to their magnitude and direction, with absolute values of approximately 0.10, 0.30, and 0.50 considered small, moderate, and strong, respectively. All tests were two-tailed, and statistical significance was set at *p* < .05. Descriptive analyses and correlation analyses were performed using IBM SPSS Statistics for Windows, Version 30.0. Internal consistency, item-rest correlations, and CFA were conducted using jamovi Version 2.5 with the SEMLj module 51. 

## Results

### Cognitive debriefing and final Swedish items

Participants generally considered the Swedish version comprehensible and relevant. However, the cognitive debriefing identified several expressions that were perceived as insufficiently idiomatic or potentially ambiguous in Swedish. In particular, the wording corresponding to “I believe” in the final item was considered overly literal and was revised to emphasize the respondent’s subjective experience of balance. The wording corresponding to “about right” in the third item was also discussed, as the initial expression was perceived as unnatural in Swedish. It was therefore replaced with a more idiomatic formulation conveying that the balance was perceived as appropriate. Participants also suggested removing temporal and summary expressions corresponding to “currently” and “overall.” These suggestions were not adopted because the expressions were considered to represent meaningful components of the original items and were retained to preserve conceptual equivalence. The introductory instruction was linguistically refined to clarify that respondents should consider both work and non-work activities, while retaining the original recall period and examples. Following a review of the cognitive debriefing findings, minor wording revisions were made, and the final Swedish version was approved after proofreading and consensus review. The translated Work-Life Balance Scale is found in Table 1.

**Table 1:** Work-life Balance Scale in Swedish

När jag reflekterar över dels mina arbetsrelaterade aktiviteter, dels mina privata aktiviteter (d.v.s. mina vardagliga aktiviteter utanför arbetet, som t.ex. familj, vänner, idrott…) under de senaste tre månaderna, så drar jag slutsatsen att…					
	Instämmer verkligen inte	Instämmer inte	Neutral	Instämmer	Instämmer verkligen
Item 1: För närvarande har jag en god balans mellan tiden jag spenderar på arbetet och tiden jag har tillgänglig för privata aktiviteter.					
Item 2: Jag har svårt att balansera mina arbetsrelaterade och privata aktiviteter.					
Item 3: Jag känner att balansen mellan mina arbetskrav och privata aktiviteter för närvarande är helt okej.					
Item 4: På det stora hela tycker jag att mitt arbetsliv och privatliv är balanserat.					

Note. For the English version of the items, see Table 3

### Psychometric evaluation

#### Participant characteristic

The total sample comprised *N* = 232 participants. Of these participants, *n* = 215 provided complete responses to all four Work-Life Balance Scale items and were included in the analysis. Hence, there are missing data for *n* = 17 persons. Police students numbered *n* = 173, and *n* = 42 were students from the prison and probation service, of whom 45% were women and 55% were men. Among the 214 participants, 32% had children living at home (Table 2).

**Table 2:** Participant characteristics of the analytic sample, *n* = 215

Police students	173 (80.5)
Prison and probation service students	42 (19.5)
Women	96 (44.7)
Men	119 (55.3)
Children living at home: Yes	69 (32.1)
	Mean (SD)
Age, years	32 (10.3)

Note. Values are presented as n (%) unless otherwise stated. Percentages are based on valid responses. Data on age and children living at home were missing for one participant each (*n* = 214).

#### Scale completion and item-level descriptive statistics

All four items used the full response range from 1 to 5 and demonstrated adequate variability. Before reverse coding, the negatively worded item had a mean score of 2.3. After reverse coding, its mean score was 3.7, as reported in Table 3. The remaining items ranged from 3.8 to 4.0. After all items had been scored in the same direction, skewness values ranged from − 0.9 to -0.6, while kurtosis values ranged from − 0.6 to 0.4 and kurtosis values ranged from − 0.56 to 0.4, indicating no substantial distributional problems at the item level.

The mean total scale score was 3.9 (SD = 0.9), with a median of 4.0. No participant obtained the lowest possible scale score. In contrast, 34 of the 215 participants (15.8%) achieved a maximum score of 5.00, indicating a modest ceiling effect. This indicates that the scale may have a somewhat limited ability to distinguish between participants at the upper end of work-life balance.

**Table 3:** Descriptive statistics, item analysis, and standardized factor loadings for the Swedish Work-Life Balance Scale

Item	Mean	SD	Skewness	Kurtosis	Corrected item-rest correlation	Standardized factor loading
1. *I currently have a good balance between the time I spend at work and the time I have available for non-work activities*	3.8	1.0	-0.8	0.2	0.75	0.88
2. *I have difficulty balancing my work and non-work activities*ᵃ	3.7	1.2	-0.6	-0.6	0.39	0.49
3. *I feel that the balance between my work demands and non-work activities is currently about right*	3.9	1.0	-0.9	0.4	0.79	0.96
4. *Overall*,* I believe that my work and non-work life are balanced*	4.0	1.0	-0.8	0.1	0.78	0.93
Total scale	**3.9**	**0.9**				

Note. ^a^. Item 2 was reverse-coded for the analyses

#### Internal consistency

The four-item Swedish Work-Life Balance Scale demonstrated good internal consistency, with Cronbach’s α = 0.83 and McDonald’s ω = 0.86. Corrected item-rest correlations were strong for items 1, 3, and 4, ranging from 0.75 to 0.79. The reverse-worded item showed a weaker but positive item-rest correlation of 0.39. Removal of this item would have increased Cronbach’s α to 0.91 and McDonald’s ω to 0.91. Inter-item correlations ranged from 0.35 to 0.83 (mean inter-item correlation = 0.57). The reverse-worded item showed consistently lower correlations with the remaining items (0.35–0.37), whereas correlations among the three positively worded items ranged from 0.73 to 0.83. See Table 4. However, the item was retained to preserve the content and structure of the original four-item scale.

**Table 4:** Inter-item correlation matrix for the Work-Life Balance Scale

	Item 1	Item 2	Item 3	Item 4
Item 1	_			
Item 2	0.37			
Item 3	0.77	0.35		
Item 4	0.73	0.36	0.83	

Note. Item 2 was reverse-coded for the analysis

#### Structural validity

A CFA was conducted to examine the proposed one-factor structure of the Swedish Work-Life Balance Scale. The four items were treated as ordered indicators, and the model was estimated using diagonally weighted least squares. The model demonstrated mixed but generally acceptable fit, with a mean-adjusted scaled chi-square of χ²(2) = 6.9, *p* = .03, a SRMR of 0.01, and RMSEA of 0.07 (95% CI [0.02, 0.14]). CFI and TLI were both 1.0. Standardized factor loadings were strong for items 1, 3, and 4, ranging from 0.88 to 0.96, whereas the reverse-worded item 2 demonstrated a lower loading of 0.49, see Table 3. Overall, the findings supported the proposed one-factor structure, though item 2 showed comparatively weaker performance, which should be noted.

#### Convergent validity and construct validity through hypothesis testing

Convergent validity was examined by assessing the association between the Swedish Work-Life Balance Scale and the OBQ-11. As hypothesized, work-life balance was strongly and positively correlated with occupational balance, *r* = .76, *p* < .001, *n* = 215, supporting convergent validity and indicating that higher perceived work-life balance is associated with higher occupational balance. Construct validity was further examined through predefined hypotheses concerning associations with symptoms of depression (PHQ-9) and anxiety (GAD-7). As hypothesized, work-life balance was negatively associated with depressive symptoms, *r* = − .54, *p* < .001, *n* = 215, and anxiety symptoms, *r* = − .45, *p* < .001, *n* = 215. These moderate negative correlations indicated that higher perceived work-life balance was associated with lower levels of depressive and anxiety symptoms, providing additional support for construct validity.

## Discussion

The aim of this study was to translate and cross-culturally validate the Work-Life Balance Scale into Swedish and to evaluate its initial psychometric properties. Overall, the findings supported the sound psychometrics of the Swedish version. The scale demonstrated good internal consistency, support for the proposed one-factor structure, strong convergent validity in relation to occupational balance, and theoretically expected negative associations with depressive and anxiety symptoms. However, the reverse-worded item performed weaker than the remaining items, and a modest ceiling effect was observed.

The small ceiling effect may indicate that the scale has a somewhat limited ability to distinguish individuals with high levels of perceived work-life balance. One possible explanation is the composition of the sample, which consisted of students in professional education rather than employees fully exposed to long-term occupational demands. Hence, the work aspect of the scale might be less strenuous, as the participants might be able to manage their time more freely than workers. Thus, the ceiling effect should therefore be examined in future studies involving employed populations, including workers exposed to shift work, high workloads, or substantial work-family demands.

The reverse-worded item showed a lower standardized factor loading (0.488) and weaker corrected item-rest correlation (0.388) than the other three items, and Cronbach’s alpha increased from 0.831 to 0.913 when the item was removed. The inter-item correlations showed the same pattern, with item 2 correlating more weakly with the remaining items (0.35–0.37) than the three positively worded items correlated with each other (0.73–0.83). The comparatively high correlations among the positively worded items may also indicate some degree of content overlap. This comparatively weak performance warrants attention, particularly because reverse-worded items may introduce wording-related method variance by requiring respondents to shift response direction and thereby increasing the risk of cognitive burden, inattentive responding, or misinterpretation [52, 53]. The fact that the same item also performed comparatively weakly in the original validation study [25] is consistent with, but does not establish, a wording-related method effect. Because item 2 is the scale’s only reverse-worded item, the present study could not distinguish such a method effect from item-specific content. Although removing the item substantially increased Cronbach’s alpha, this finding alone does not justify adopting a three-item version, as doing so would alter the original measure and the resulting version has not been independently validated. Until further evidence is available, Swedish users should therefore retain all four items and the original scoring to preserve comparability, while reporting item-level performance and interpreting item 2 cautiously. However, future studies could directly compare the reverse-worded item with a semantically equivalent positively worded version and examine whether rewording improves reliability, factor loading, and validity without reducing construct coverage. Item-level functioning should also be examined in larger and more occupationally diverse samples using item response theory, which could clarify whether all four items contribute distinct information across the work-life balance continuum.

The CFA provided initial, although mixed, support for the original one-factor structure. Some fit indices indicated good model fit, whereas the scaled chi-square test and RMSEA suggested some degree of misfit. These mixed findings should be interpreted in light of the short scale and the model’s low degrees of freedom, as RMSEA may be unstable in models with few degrees of freedom [54]. Nevertheless, three of the four items showed strong standardized factor loadings, supporting the interpretation of the scale as primarily unidimensional and aligning with the original study [25].

The scale demonstrated evidence of convergent validity through its strong positive association with occupational balance. Individuals reporting higher work-life balance also tended to report a higher degree of balance among their everyday activities. This finding is theoretically plausible because both constructs concern perceived balance in daily life. However, the magnitude of the correlation (*r* = .762) also indicates substantial conceptual and empirical overlap and raises questions about the extent to which the two instruments capture distinct constructs. Occupational balance encompasses the broader pattern, amount, and variation of everyday activities, whereas work-life balance focuses more specifically on the relationship between work and non-work activities. The Work-Life Balance Scale may therefore be particularly useful when the research question concerns this specific relationship or when a brief measure is required, whereas the OBQ-11 provides a broader assessment of balance across everyday occupations. Nevertheless, the present study did not examine whether the Work-Life Balance Scale provides incremental information beyond the OBQ-11. Future studies should therefore examine the discriminant and incremental validity of the two measures. Furthermore, construct validity was supported by the theoretically expected negative associations with depressive and anxiety symptoms measured with PHQ-9 and GAD-7. Participants reporting higher work-life balance had lower PHQ-9 and GAD-7 scores. These findings are consistent with the original validation study, in which higher work-life balance was associated with lower psychological strain [25]. The findings are also in line with prior studies on how experiencing both work-life balance [55, 56] and occupational balance [57, 58] across different professions is associated with good health. On the contrary, imbalances in life may cause mental ill-health. Thus, the present study extends the evidence of the Work-Life Balance Scale by demonstrating associations with more specific indicators of depressive and anxiety symptoms. However, the cross-sectional design means the direction of these relationships cannot be established. Hence, causal relationships also need further investigation.

### Strengths and limitations

A key strength of the study was the systematic translation and cross-cultural adaptation process, which included independent forward translations, independent back translations, reconciliation, cognitive debriefing, and final review. The inclusion of cognitive debriefing provided evidence that the Swedish wording was understandable and culturally appropriate. However, the cognitive debriefings might have benefited from including participants from a wider variety of occupations.

Another strength of the study was the use of several complementary psychometric analyses, including CFA, Cronbach’s alpha, McDonald’s omega, corrected item-rest correlations, convergent validity, and hypothesis-based construct validity. The use of both occupational balance and psychological health measures also allowed the scale to be evaluated in relation to theoretically relevant but distinct constructs. However, a few limitations should be considered regarding the psychometric analyses. First, the sample consisted of police students and students from the prison and probation service. Although the student groups are relevant to future research on public emergency and security personnel, they do not represent the broader Swedish working population. The findings may therefore not generalize to workers in other sectors, age groups, or employment conditions. However, the participants’ relatively high mean age and family responsibilities suggest that many may have had prior experience of combining work and non-work roles. Regardless, we did not collect information on employment history and occupational background, which limits conclusions about the applicability of the findings to working populations. In addition, the predominance of participants from police- and probation-related education may limit the generalizability of the psychometric properties observed here, highlighting the need for further validation in more occupationally diverse samples. Second, the cross-sectional design did not allow for the assessment of test-retest reliability, responsiveness, temporal stability, or predictive validity. Longitudinal studies are needed to determine whether the Swedish scale predicts later psychological health, work ability, sickness absence, job satisfaction, or turnover intentions. Third, the CFA was based on a relatively modest sample and a model with only four items and two degrees of freedom. This limited the precision and stability of some fit indices, particularly RMSEA. The one-factor structure should therefore be replicated in larger and more heterogeneous samples. Lastly, the translation process and cross-cultural validation did not include bilingual persons, or included a translation agency, which could have provided an objective translation service. Despite the thorough translation process, translator-related bias may have influenced the translation of the instrument.

## Conclusions

The Swedish version of the Work-Life Balance Scale demonstrated promising initial psychometric properties, consistent with the original Work-Life Balance Scale study. Hence, the Swedish Work-Life Balance Scale will be useful in research and practice when a measure of perceived balance between work and non-work activities is required. Its short format reduces respondent burden and may facilitate inclusion in broader surveys of health, work ability, and working conditions. Further evaluation is needed before conclusions can be drawn regarding its performance across different occupational groups and over time. Future studies should, e.g., evaluate test-retest reliability, measurement invariance, responsiveness, and predictive validity. Given the brevity of the four-item scale, future research using Item Response Theory (IRT) in larger and more occupationally diverse working samples could complement the present classical test theory findings by providing more detailed information on individual item functioning, discrimination, and measurement precision across different levels of work–life balance.

## Data Availability

The data that support the findings of this study are available from Elin Granholm Valmari, but restrictions apply to their availability; they were used under license for the current study and are therefore not publicly available. Data are, however, available from the authors upon reasonable request and with permission of the Swedish Ethical Review Authority.

## Abbreviations

CFA: Confirmatory Factor Analysis
CFI: Comparative Fit Index
DWLS: Diagonally Weighted Least Squares
GAD: 7-The Generalized Anxiety Disorder-7
ISPOR: International Society for Pharmacoeconomics and Outcomes Research
OBQ: 11-Occupational Balance Questionnaire
PHQ: 9-Patient Health Questionnaire-9
REDCap: Research Electronic Data Capture
RMSEA: Root Mean Square Error of Approximation
SEMlj: Structural Equation Modeling module for jamovi
SPSS: Statistical Package for the Social Sciences
SRMR: Standardized Root Mean Square Residual
TLI: Tucker-Lewis Index

## References

[1] Aronsson G, Theorell T, Grape T, Hammarström A, Hogstedt C, Marteinsdottir I, Skoog I, Träskman-Bendz L, Hall C. A systematic review including meta-analysis of work environment and burnout symptoms. BMC Public Health. 2017;17(1):1–13. 10.1186/s12889-017-4153-7. PMC5356239 28302088

[2] Ahlstrom L, Grimby-Ekman A, Hagberg M, Dellve L. The work ability index and single-item question: Associations with sick leave, symptoms, and health – a prospective study of women on long-term sick leave. Scand J Work Environ Health. 2010;36(5):404–12. 10.5271/sjweh.2917. 20372766

[3] Tuomi K, Ilmarinen J, Eskelinen L, Järvinen E, Toikkanen J, Klockars M. (2017). *Work Ability Index* [Dataset]. 10.1037/t58607-000

[4] Abendroth A-K, den Dulk L. Support for the work-life balance in Europe: The impact of state, workplace and family support on work-life balance satisfaction. Work Employ Soc. 2011;25(2):234–56.

[5] Thilagavathy S, Geetha SN. A morphological analyses of the literature on employee work-life balance. Curr Psychol. 2020;2003 10.1007/s12144-020-00968-x.

[6] Nilsen W, Skipstein A, Østby KA, Mykletun A. Examination of the double burden hypothesis-a systematic review of work-family conflict and sickness absence. Eur J Pub Health. 2017;27(3):465–71. 10.1093/eurpub/ckx054. PMC5445721 28486653

[7] Çivilidağ A, Durmaz Ş. The relationship of flexible working arrangements on work-family conflict, work-life balance and organizational commitment: A systematic review and meta-analysis. BMC Psychol. 2026;14(1):472. 10.1186/s40359-026-04216-y. PMC13059400 41764591

[8] Nurizka MS, Wahyuningsih SH, Rahayu M. Work Life Balance (WLB) and Job Satisfaction (JS): A Systematic Literature Review. Calitatea. 2024;25(203):244–56. 10.47750/QAS/25.203.26.

[9] Panda A, Sahoo CK. Work–life balance, retention of professionals and psychological empowerment: An empirical validation. Eur J Manage Stud. 2021;26(2/3):103–23. 10.1108/EJMS-12-2020-0003.

[10] Fan Y, Potočnik K, Chaudhry S. A process-oriented, multilevel, multidimensional conceptual framework of work–life balance support: A multidisciplinary systematic literature review and future research agenda. Int J Manage Reviews: IJMR. 2021;23(4):486–515. 10.1111/ijmr.12254.

[11] Rashmi K, Kataria A. Work–life balance: A systematic literature review and bibliometric analysis. Int J Sociol Soc Policy. 2022;42(11/12):1028–65. 10.1108/IJSSP-06-2021-0145.

[12] Khateeb FR. WORK LIFE BALANCE - A REVIEW OF THEORIES, DEFINITIONS AND POLICIES. Cross-Cultural Manage J. 2021;XXIII(1):27–55.

[13] Greenhaus JH, Beutell NJ. Sources of conflict between work and family roles1. Acad Manage Acad Manage Rev (Pre-1986). 1985;10(000001):76.

[14] Amstad FT, Meier LL, Fasel U, Elfering A, Semmer NK. (2011). A meta-analysis of work–family conflict and various outcomes with a special emphasis on cross-domain versus matching-domain relations. *Journal of Occupational Health Psychology*, *16*(2), 151–169. (2011-01608-001). 10.1037/a0022170 21280939

[15] Greenhaus JH, Powell GN. WHEN WORK AND FAMILY ARE ALLIES: A THEORY OF WORK-FAMILY ENRICHMENT. Acad Manage Rev. 2006a;31(1):72–9219379625. 10.5465/AMR.2006.19379625.

[16] Greenhaus JH, Powell GN. When Work and Family Are Allies: A Theory of Work-Family Enrichment. Acad Manage Rev. 2006b;31(1):72–92. 10.2307/20159186.

[17] Bakker AB, Demerouti E. The spillover-crossover model. In: Grzywacz J, Demerouti E, editors. New frontiers in work and family research. 1st ed. Psychology; 2013. pp. 54–70.

[18] Grzywacz J, Demerouti E. New Frontiers in Work and Family Research. Taylor & Francis Group; 2013. http://ebookcentral.proquest.com/lib/umeaub-ebooks/detail.action?docID=1143868.

[19] Kalliath T, Brough P. Work–life balance: A review of the meaning of the balance construct. J Manage Organ. 2008;14(3):323–7. 10.5172/jmo.837.14.3.323.

[20] Perreault M, Power N. Work-life balance as a personal responsibility: The impact on strategies for coping with interrole conflict. J Occup Sci. 2023;30(2):160–74. 10.1080/14427591.2021.1894596.

[21] Håkansson C, Wagman P, Hagell P. Construct validity of a revised version of the Occupational Balance Questionnaire. Scand J Occup Ther. 2020;27(6):441–9. 10.1080/11038128.2019.1660801. 31524026

[22] Wagman P, Håkansson C, Björklund A. Occupational balance as used in occupational therapy: A concept analysis. Scand J Occup Ther. 2012;19(4):322–7. 10.3109/11038128.2011.596219. 21780985

[23] Alameddine M, Al-Yateem N, Bou-Karroum K, Hijazi H, Al Marzouqi A, Al-Adawi S. Measurement of Work-Life Balance: A Scoping Review with a Focus on the Health Sector. J Nurs Adm Manag. 2023;2023:1–10. 10.1155/2023/3666224. PMC11919065 40225624

[24] Rizzo A, YILDIRIM M, KHABBACHE MAGGIOMG, GOMEZ-SALGADO H, Mahmood J, Driss BAHRAMIZADEH, Lukasz AITALI, Pietro SZARPAK, Giada CRESCENZO, Nicola IUELE, Livio BRAGAZZI TARCHI, Francesco CHIRICO. Novel measures to assess work-life balance: A systematic review of last 5 years (2018–2023). J Health Social Sci. 2023;8(4):270–81. 10.19204/2023/NVLM2.

[25] Brough P, Timms C, O’Driscoll MP, Kalliath T, Siu O-L, Sit C, Lo D. Work–life balance: A longitudinal evaluation of a new measure across Australia and New Zealand workers. Int J Hum Resource Manage. 2014;25(19):2724–44. 10.1080/09585192.2014.899262.

[26] Liswandi L, Muhammad R. The Association Between Work-life Balance and Employee Mental Health: A systemic review. Asia Pac J Health Manage. 2023;18(3):263–80. 10.24083/apjhm.v18i3.2565.

[27] Haar J, Harris C, Myers B. The effects of work-life balance on the well-being of older workers: Same-same or same-different? Int J Manpow. 2024;45(3):484–99. 10.1108/IJM-04-2022-0181.

[28] Adedeji A, Langel C, Feick A, Greibaum M-SB, Rahimi M, Hanft-Robert S. Work-life balance and mental health outcomes for Generation Z in Germany. J Occup Environ Med. 2023;65(12):987–91. 10.1097/JOM.0000000000002934. 37505083

[29] Sprung JM, Rogers A. Work-life balance as a predictor of college student anxiety and depression. J Am Coll Health. 2021;69(7):775–82. 10.1080/07448481.2019.1706540. 31971892

[30] Wild D, Grove A, Martin M, Eremenco S, McElroy S, Verjee-Lorenz A, Erikson P. Principles of Good Practice for the Translation and Cultural Adaptation Process for Patient-Reported Outcomes (PRO) Measures: Report of the ISPOR Task Force for Translation and Cultural Adaptation. Value Health. 2005;8(2):94–104. 10.1111/j.1524-4733.2005.04054.x. 15804318

[31] Wagman P, Håkansson C. Introducing the Occupational Balance Questionnaire (OBQ). Scand J Occup Ther. 2014;21(3):227–31. 10.3109/11038128.2014.900571. 24649971

[32] Günal A, Pekçetin S, Demirtürk F, Şenol H, Håkansson C, Wagman P. Validity and reliability of the Turkish Occupational Balance Questionnaire (OBQ11-T). Scand J Occup Ther. 2020;27(7):493–9. 10.1080/11038128.2019.1673479. 31608736

[33] Salehi sadati SS, Raji P, Nakhostin Ansari N, Håkansson C, Wagman P. Translation, cultural adaptation and psychometric properties of the Persian version of Occupational Balance Questionnaire in healthy adults. Br J Occup Therapy. 2023;86(8):587–94. 10.1177/03080226231174103. PMC12033656 40337612

[34] Uhrmann L, Hovengen I, Wagman P, Håkansson C, Bonsaksen T. The Norwegian Occupational Balance Questionnaire (OBQ11-N) – development and pilot study. Scand J Occup Ther. 2019;26(7):546–51. 10.1080/11038128.2018.1523458. 30422027

[35] Kroenke K, Spitzer RL, Williams JBW. (2011). *Patient Health Questionnaire-9* [Dataset]. 10.1037/t06165-000

[36] Adler M, Hetta J, Isacsson G, Brodin U. An item response theory evaluation of three depression assessment instruments in a clinical sample. BMC Med Res Methodol. 2012;12:84. 10.1186/1471-2288-12-84. PMC3599629 22721257

[37] Hansson M, Chotai J, Nordstöm A, Bodlund O. Comparison of two self-rating scales to detect depression: HADS and PHQ-9. Br J Gen Pract. 2009;59(566):e283–8. 10.3399/bjgp09X454070. PMC2734374 19761655

[38] Hlynsson JI, Gustafsson O, Carlbring P. Uncertainty Breeds Anxiety and Depression: The Impact of the Russian Invasion in Ukraine on a Swedish Clinical Population Receiving Internet-Based Psychotherapy. Clin Psychol Europe. 2024;6(1):1–20. 10.32872/cpe.12083. PMC11303911 39119223

[39] Swedish Agency for Health Technology Assessment and Assessment of Social Services. (2018). *Patient Health Questionnaire-9 (PHQ-9) som stöd för diagnostik och bedömning av svårighetsgrad av depression* (Systematisk litteraturöversikt; HTA; Systematic review No. 276; SBU Utvärderar). Swedish Agency for Health Technology Assessment and Assessment of Social Services. https://www.sbu.se/sv/publikationer/SBU-utvarderar/patient-health-questionnaire-9-phq-9-som-stod-for-diagnostik-och-bedomning-av-svarighetsgrad-av-depression/

[40] Costantini L, Pasquarella C, Odone A, Colucci ME, Costanza A, Serafini G, Aguglia A, Belvederi Murri M, Brakoulias V, Amore M, Ghaemi SN, Amerio A. Screening for depression in primary care with Patient Health Questionnaire-9 (PHQ-9): A systematic review. J Affect Disord. 2021;279:473–83. 10.1016/j.jad.2020.09.131. 33126078

[41] Spitzer RL, Kroenke K, Williams JBW, Löwe B. A brief measure for assessing generalized anxiety disorder: The GAD-7. Arch Intern Med. 2006;166(10):1092–7. 10.1001/archinte.166.10.1092. 16717171

[42] Swedish Association of Local Authorities and Regions. (n.d.). The Swedish National Care and Intervention Programmes. Retrieved September 25. 2026, from https://www.vardochinsats.se/depression-och-aangestsyndrom/

[43] Ahlqvist Lindqvist E, Johansson M, Hovén E, Frick A, Ljungvall H, Åsenlöf P. Psychometric assessment and exploratory scale refinement of the Generalized Anxiety Disorder 7-item scale among adolescents and young adults in a Swedish context. BMC Psychiatry. 2026;26(1):564. 10.1186/s12888-026-08423-0. PMC13397768 42498940

[44] Plummer F, Manea L, Trepel D, McMillan D. Screening for anxiety disorders with the GAD-7 and GAD-2: A systematic review and diagnostic metaanalysis. Gen Hosp Psychiatry. 2016;39:24–31. 10.1016/j.genhosppsych.2015.11.005. 26719105

[45] Tiirikainen K, Haravuori H, Ranta K, Kaltiala-Heino R, Marttunen M. Psychometric properties of the 7-item Generalized Anxiety Disorder Scale (GAD-7) in a large representative sample of Finnish adolescents. Psychiatry Res. 2019;272:30–5. 10.1016/j.psychres.2018.12.004. 30579178

[46] Todorović A, Baumann C, Blanchin M, Bourion-Bédès S. Validation of Generalized Anxiety Disorder 6 (GAD-6)-A Modified Structure of Screening for Anxiety in the Adolescent French Population. Int J Environ Res Public Health. 2023;20(8):5546. 10.3390/ijerph20085546. PMC10138941 37107826

[47] Terwee CB, Bot SD, de Boer MR, van der Windt DA, Knol DL, Dekker J, Bouter LM, de Vet HC. Quality criteria were proposed for measurement properties of health status questionnaires. J Clin Epidemiol. 2007;60(1):34–42. 10.1016/j.jclinepi.2006.03.012. 17161752

[48] Cronbach LJ. Coefficient Alpha and the Internal Structure of Tests. Psychometrika. 1951;16(3):297–334. 10.1007/BF02310555.

[49] McDonald RP. Test theory: A unified treatment. 1st ed. L. Erlbaum Associates; 1999. 10.4324/9781410601087.

[50] Kalkbrenner MT. Choosing Between Cronbach’s Coefficient Alpha, McDonald’s Coefficient Omega, and Coefficient H: Confidence Intervals and the Advantages and Drawbacks of Interpretive Guidelines. Meas Evaluation Couns Dev. 2024;57(2):93–105. 10.1080/07481756.2023.2283637.

[51] *The jamovi project* (Version 2.5.6). (2025). [Jamovi]. https://www.jamovi.org

[52] Hughes GD. (2009). The Impact of Incorrect Responses to Reverse-Coded Survey Items. *Research in the Schools*, *16*(2), 76–88. (49314586).

[53] Weijters B, Baumgartner H. Misresponse to Reversed and Negated Items in Surveys: A Review. J Mark Res. 2012;49(5):737–47. 10.1509/jmr.11.0368.

[54] Kenny DA, Kaniskan B, McCoach DB. The Performance of RMSEA in Models With Small Degrees of Freedom. Sociol Methods Res. 2015;44(3):486–507. 10.1177/0049124114543236.

[55] Saroj P. Work-Life Balance and Mental Health: A Psychological Study of Professional Women in High-Stress Occupations. Int J Res Sci Innov. 2025;XII(V):1617–25. 10.51244/IJRSI.2025.120500154.

[56] Zhang J, Rehman S, Addas A, Ahmad J. Influence of Work-Life Balance on Mental Health Among Nurses: The Mediating Role of Psychological Capital and Job Satisfaction. Psychol Res Behav Manage. 2024;17:4249–62. 10.2147/PRBM.S497305. PMC11646404 39679316

[57] Huertas-Hoyas E, García-Bravo C, Pérez-Corrales J, Bullón-Benito E, Fernández-Gómez G, Rodríguez-Pérez MP. Association between occupational balance and the physical and mental health of the university community: An observational study. Front Public Health. 2025;13:1631096. 10.3389/fpubh.2025.1631096. PMC12350463 40823247

[58] Marín-Berges M, Lizana PA, Marroquín-Pinochet V, Osorio-Paredes V, Iguacel I, Gómez-Bruton A. Mental health, quality of life, and occupational balance among taxi drivers in Spain and Chile: A cross-cultural study. J Transp Health. 2025;44:102105. 10.1016/j.jth.2025.102105.

